# Synthesis and characterisation of celastrol derivatives as potential anticancer agents

**DOI:** 10.1080/14756366.2017.1404590

**Published:** 2017-12-12

**Authors:** Hong-Jian Zhang, Guo-Rui Zhang, Hu-Ri Piao, Zhe-Shan Quan

**Affiliations:** Key Laboratory of Natural Resources and Functional Molecules of the Changbai Mountain, Affiliated Ministry of Education, College of Pharmacy, Yanbian University, Yanji, Jilin, China

**Keywords:** Synthesis, celastrol, amino acid, triazole, anticancer, docking

## Abstract

In the present study, three series of novel celastrol derivatives were designed and synthesised by modifying the carboxylic acid at the 20th position with amino acid, amine, and triazole derivatives. All the synthesised compounds were screened for their anticancer activities using MTT assay against AGS, MGC-803, SGC-7901, HCT-116, A549, HeLa, BEL-7402, and HepG-2 cell lines. Most of the synthesised compounds exhibited potent antiproliferative effects. The most promising compound 3-Hydroxy-9*β*,13*α*-dimethyl-2-oxo-24,25,26-trinoroleana-1(10),3,5,7-tetraen-29-oic amide, *N*-(*R*)-methyl-3-(1*H*-indol-2-yl)propanoate (**11**) showed considerable high anticancer activity against AGS cell lines, with an IC_50_ value of 0.44 μM, and considerably higher activities against HCT-116, BEL-7402, and HepG-2 cell lines, with IC_50_ values of 0.78, 0.63, and 0.76 μM, respectively. The results of apoptosis tests and molecular docking study of compound **11** binding to Caspase-3 revealed that its mechanism of action with antiproliferative was possibly involved in inducing apoptosis by inducing the activation of caspase-3.

## Introduction

Celastrol, a quinone methide triterpene, is an active ingredient first extracted from the roots of the Chinese medicinal plant “Thunder of God Vine” (Celastraceae, Tripterygium). A literature survey showed that celastrol can destroy cell lines by activating the classical apoptotic pathway, and it strongly inhibits cell proliferation. It was proven to have several biological activities, such as anticancer^1–4^, antifungal^5^, antifibrotic^6^, anti-inflammatory^7^^,^^8^, and antioxidant^6^^,^^9^ activities, and resistance to neurodegenerative disease activity^10^. Although celastrol has important pharmacological activities, it also has several drawbacks such as poor water solubility, high toxicity, poor stability, etc., which restrict its clinical application. Thus, it is very important to improve its solubility and stability and reduce its toxicity by modifying its structure. Previous studies have also suggested that the 20th carboxylic acid position of celastrol can be easily modified^11–14^, and the derivatives play an important role in anticancer activity.

Amino acids, the basic biological and functional units of proteins, play an important role in human metabolism. Amino acid derivatives display diverse biological functions, including immunomodulatory^15^, antituberculosis^16^, antifatigue^17^, bactericidal^18^, and anticancer^19^^,^^20^ activities. In general, the solubility of compounds is directly related to the number of polar groups present, such as hydroxyl, carboxyl, amino, amide, etc. A change in activity may be followed by a change in solubility of compounds. Moreover, an amide group would increase the stability of the compound *in vitro and in vivo*, and thus, it would be less susceptible to enzymatic hydrolysis and act as a hydrogen donor. As reported by Csuk et al., compounds containing a triazole moiety bind to a variety of enzymes and receptors by non-covalent interactions; these compounds also have a wide array of biological activities^21^. In the present study, to develop novel celastrol derivatives with potent antitumor bioactivities, we introduced three different groups to the 20th carboxylic acid position of celastrol. We hypothesised that the combination of appropriate amino acids ester, amino, and amide groups with a celastrol core may have increased anticancer activity. In the present study, three series of celastrol derivatives were synthesised, and their antitumor activities were evaluated against eight cancer cell lines (AGS, MGC-803, HCT-116, SGC-7901, BEL-7402, A549, HeLa, and HepG-2). Next, to investigate its possible mechanism of action, the antiproliferative activity of the derivative with the strongest antitumor activity was evaluated.

## Experimental

### Materials and methods

All reagents were obtained commercially and were used without further purification. Solvents were dried according to standard procedures. Reactions were monitored by thin-layer chromatography (TLC) on silica gel plates. Melting points were determined in open capillary tubes and were uncorrected. ^1^H NMR and ^13^C NMR spectra were measured on an AV-300 (Bruker, Switzerland), and all chemical shifts were given in ppm relative to TMS. Mass spectra were measured on a HP1100LC (Agilent Technologies, USA). High resolution mass spectra were measured on an MALDI-TOF/TOF mass spectrometer (Bruker Daltonik, Germany).

#### General procedure for the synthesis of compounds **1**–**11**

A mixture of celastrol (45 mg, 0.1 mmol), EDC·HCl (43 mg, 0.22 mmol), HOBt (30 mg, 0.22 mmol), TEA (102 μL, 0.3 mmol), and various amino acid esters (0.3 mmol) in CH_2_Cl_2_ (5.0 ml) was stirred at 0 °C for 8 h. Next, the mixture was washed thrice with water and saline, dried using anhydrous MgSO_4_, filtered, and concentrated *in vacuo*. The mixture was concentrated and purified by normal-phase column chromatography (dichloromethane:methanol =100:1) to obtain the target compounds **11**.

#### 3-Hydroxy-9β,13α-dimethyl-2-oxo-24,25,26-trinoroleana-1(10),3,5,7-tetraen-29-oicamide,N-methyl acetate (1)

Red solid; yield, 47%; m.p. 104–106 °C; ^1 ^H NMR (300 MHz, CDCl_3_), *δ*: 7.02 (d, *J* = 7.4 Hz, 1 H), 6.54 (s, 1 H), 6.35 (d, *J* = 6.9 Hz, 1 H), 6.29 (s, 1 H), 3.94 (s, 2 H), 3.75 (s, 3 H), 2.47 (d, *J* = 15.5 Hz, 1 H), 2.22 (s, 3 H), 1.45–1.88 (m, 14 H), 1.45 (s, 3 H), 1.28 (s, 3 H), 1.20 (s, 3 H), 1.15 (s, 3 H), 0.87 (s, 1 H), 0.59 (s, 3 H). ^13 ^C NMR (75 MHz, CDCl_3_),* δ*: 178.34, 177.87, 170.82, 170.17, 164.76, 146.01, 139.95, 127.41, 119.54, 118.04, 116.99, 52.42, 45.06, 44.37, 42.98, 41.32, 40.33, 39.35, 38.20, 36.33, 34.85, 33.55, 33.48, 31.59, 31.20, 30.80, 30.04, 29.43, 28.66, 21.74, 18.15, 10.24. ESI-HRMS (*m/z*): calcd. for C_32_H_44_NO_5_^+^ [M + H]^+^: 522.3214; found: 522.3206. Purity: 100% by HPLC (A: H_2_O; B: acetonitrile, graded: 50–100%), *t*_R_ 12.947 min, λ: 400 nm.

#### 3-Hydroxy-9β,13α-dimethyl-2-oxo-24,25,26-trinoroleana-1(10),3,5,7-tetraen-29-oicamide,N-(R)-ethyl 2-propanoate (2)

Red powder; yield, 55%; m.p. 102–104 °C; ^1 ^H NMR (300 MHz, CDCl_3_), *δ*: 7.04–6.96 (m, 1 H), 6.54 (s, 1 H), 6.41 (d, *J* = 6.5 Hz, 1 H), 6.36 (s, 1 H), 4.48–4.38 (m, 1 H), 4.08–4.29 (m, 2 H), 2.46 (d, *J* = 15.3 Hz, 1 H), 2.22 (s, 3 H), 1.60–2.06 (m, 14 H), 1.45 (s, 3 H), 1.35 (d, *J* = 6.8 Hz, 3 H), 1.26 (d, *J* = 7.7 Hz, 6 H), 1.17 (d, *J* = 13.5 Hz, 6 H), 0.88 (m, 1 H), 0.61 (s, 3 H). ^13 ^C NMR (75 MHz, CDCl_3_), *δ*: 178.35, 177.26, 173.51, 170.24, 164.74, 146.01, 134.03, 127.37, 119.53, 118.03, 117.02, 61.55, 50.88, 47.91, 45.05, 44.27, 39.34, 38.14, 36.30, 34.75, 33.66, 33.45, 31.60, 30.93, 30.80, 29.96, 29.70, 29.44, 28.69, 21.78, 18.55, 18.22, 14.04, 10.25. ESI-HRMS (*m/z*): calcd for C_34_H_48_NO_5_^+^ [M + H]^+^: 550.3527; found: 550.3511. Purity: 99.427% by HPLC (A: H_2_O; B: acetonitrile, graded: 50–100%), *t*_R_ 15.047 min, λ: 400 nm.

#### 3-Hydroxy-9β,13α-dimethyl-2-oxo-24,25,26-trinoroleana-1(10),3,5,7-tetraen-29-oicamide,N-methyl 3-methyl butyrate (3)

Red powder; yield, 26%; m.p. 96–98 °C; ^1 ^H NMR (300 MHz, CDCl_3_), *δ*: 7.00 (d, *J* = 7.0 Hz, 1 H), 6.52 (s, 1 H), 6.32 (t, *J* = 7.0 Hz, 2 H), 4.42 (dd, *J* = 8.3, 4.6 Hz, 1 H), 3.67 (s, 3 H), 2.51 (d, *J* = 12.9 Hz, 1 H), 2.20 (s, 3 H), 2.10 (m, 1 H), 1.64–1.91 (m, 14 H), 1.42 (s, 3 H), 1.25–1.13 (m, 9 H), 0.97 (d, *J* = 6.1 Hz, 1 H), 0.87 (dd, *J* = 6.7, 3.5 Hz, 6 H), 0.53 (s, 3 H). ^13 ^C NMR (75 MHz, CDCl_3_), *δ*: 178.36, 177.76, 172.90, 170.20, 164.77, 146.01, 134.01, 127.38, 119.49, 118.01, 116.99, 56.69, 52.16, 45.07, 44.25, 43.02, 40.66, 39.28, 38.09, 36.27, 34.77, 33.98, 33.35, 31.58, 30.82, 30.65, 30.11, 29.34, 28.66, 21.83, 18.85,18.05, 17.82, 10.24. ESI-HRMS (*m/z*): calcd. for C_35_H_40_NO_5_^+^ [M + H]^+^: 564.3684; found: 564.3680. Purity: 98.056% by HPLC (A: H_2_O; B: acetonitrile, graded: 50–100%), *t*_R_ 16.247 min, λ: 423 nm.

#### 3-Hydroxy-9β,13α-dimethyl-2-oxo-24,25,26-trinoroleana-1(10),3,5,7-tetraen-29-oic amide, N-methyl4-methylpentanoate (4)

Red powder; yield, 29%; m.p. 102–104 °C; ^1 ^H NMR (300 MHz, CDCl_3_), *δ*: 7.02 (d, *J* = 7.1 Hz, 1 H), 6.53 (s, 1 H), 6.35 (d, *J* = 7.2 Hz, 1 H), 6.19 (d, *J* = 8.1 Hz, 1 H), 4.55 (s, 1 H), 3.74 (s, 1 H) 3.68 (s, 3 H), 2.50 (d, *J* = 13.9 Hz, 1 H), 2.23 (s, 3 H), 1.51–1.85 (m, 16 H), 1.45 (s, 3 H), 1.27 (s, 3 H), 1.19 (s, 3 H), 1.14 (s, 3 H), 0.96 (d, *J* = 5.1 Hz, 1 H), 0.92 (d, *J* = 6.0 Hz, 6 H), 0.58 (s, 3 H). ^13 ^C NMR (75 MHz, CDCl_3_), *δ*: 178.34, 177.57, 173.92, 170.15, 164.78, 145.99, 133.98, 127.38, 119.44, 118.03, 117.00, 52.27, 50.41, 45.05, 44.29, 43.00, 42.00, 40.50, 39.27, 38.13, 36.29, 34.79, 33.79, 33.32, 31.63, 30.79, 30.75, 30.08, 29.41, 28.65, 24.87, 22.91, 22.12, 21.85, 18.14, 10.26. ESI-HRMS (*m/z*): calcd. for C_36_H_52_NO_5_^+^ [M + H]^+^: 578.3840; found: 578.3846. Purity: 100% by HPLC (A: H_2_O; B: acetonitrile, graded: 50–100%), *t*_R_ 19.153 min, λ: 423 nm.

#### 3-Hydroxy-9β,13α-dimethyl-2-oxo-24,25,26-trinoroleana-1(10),3,5,7-tetraen-29-oic amide, N-methyl 4-(methylthio)butanoate (5)

Red powder; yield, 38%; m.p. 116–118 °C; ^1 ^H NMR (300 MHz, CDCl_3_), *δ*: 7.02 (d, *J* = 7.1 Hz, 1 H), 6.76 (s, 1 H), 6.66 (d, *J* = 6.5 Hz, 1 H), 6.54 (s, 1 H), 6.35 (d, *J* = 7.5 Hz, 1 H), 4.55 (m, 1 H), 3.76 (s, 2 H), 3.73 (d, *J* = 4.6 Hz, 3 H), 2.66 (d, *J* = 7.8 Hz, 1 H), 2.47 (m, 3 H), 2.22 (s, 3 H), 2.17 (s, 2 H), 2.09 (s, 3 H), 1.96–1.61 (m, 12 H), 1.45 (s, 3 H), 1.27 (s, 3 H), 1.21–1.14 (m, 6 H), 0.88 (d, *J* = 6.4 Hz, 1 H), 0.57 (s, 3 H).^13 ^C NMR (75 MHz, CDCl_3_), *δ*: 178.32, 177.82, 172.78, 170.19, 164.73, 146.01, 134.02, 127.39, 119.53, 118.05, 117.03, 52.56, 51.47, 45.06, 44.26, 43.01, 40.51, 39.31, 38.15, 36.58, 36.28, 34.74, 33.80, 33.39, 31.61, 31.33, 30.81, 30.72, 29.94, 29.41, 28.65, 21.80, 18.15, 15.56, 10.26. ESI-HRMS (*m/z*): calcd for C_35_H_50_NO_5_S^+^ [M + H]^+^: 596.3404; found: 596.3412. Purity: 100% by HPLC (A: H_2_O; B: acetonitrile, graded: 50–100%), *t*_R_ 15.407 min, λ: 400 nm.

#### 3-Hydroxy-9β,13α-dimethyl-2-oxo-24,25,26-trinoroleana-1(10),3,5,7-tetraen-29-oic amide, N-(R)-methyl -2-phenylacetate (6)

Red powder; yield, 57%; m.p. 144–146 °C; ^1 ^H NMR (300 MHz, CDCl_3_), *δ*: 7.27 (d, *J* = 2.2 Hz, 4 H), 6.99 (d, *J* = 15.7 Hz, 2 H), 6.69 (s, 1 H), 6.47 (s, 1 H), 6.30 (s, 1 H), 5.48 (d, *J* = 7.1 Hz, 1 H), 3.67 (s, 3 H), 2.32 (s, 1 H), 2.24 (d, *J* = 15.8 Hz, 3 H), 1.90–1.52 (m, 10 H), 1.39 (s, 3 H), 1.21 (s, 3 H), 1.16 (s, 3 H), 1.10 (s, 3 H), 0.86 (m, 4 H), 0.79 (s, 1 H), 0.41 (s, 3 H). ^13 ^C NMR (75 MHz, CDCl_3_), *δ*: 178.29, 177.04, 171.90, 170.23, 164.69, 145.98, 136.65, 134.01, 129.07, 128.60, 127.34, 127.25, 119.60, 117.99, 117.09, 55.87, 52.81, 44.99, 44.35, 42.87, 40.13, 39.24, 38.18, 36.29, 34.70, 33.57, 33.11, 31.55, 31.32, 30.75, 29.98, 29.37, 28.61, 21.69, 18.11, 10.27. ESI-HRMS (*m/z*): calcd for C_38_H_48_NO_5_^+^ [M + H]^+^: 598.3527; found: 598.3513. Purity: 100% by HPLC (A: H_2_O; B: acetonitrile, graded: 50–100%), *t*_R_ 16.447 min, λ: 400 nm.

#### 3-Hydroxy-9β,13α-dimethyl-2-oxo-24,25,26-trinoroleana-1(10),3,5,7-tetraen-29-oic amide, N-(R)-methyl 3-phenylpropanoate (7)

Red powder; yield, 61%; m.p. 128–130 °C; ^1 ^H NMR (300 MHz, CDCl_3_), *δ*: 7.11 (m, 5 H), 6.99 (s, 1 H), 6.53 (s, 1 H), 6.34 (d, *J* = 7.1 Hz, 1 H), 6.07 (s, 1 H), 4.63 (d, *J* = 6.2 Hz, 1 H), 3.71 (s, 3 H), 3.03 (dd, *J* = 14.3, 5.8 Hz, 2 H), 2.34 (s, 1 H), 2.24 (s, 3 H), 2.06 (d, *J* = 20.3 Hz, 2 H), 1.66 (dd, *J* = 53.8, 19.6 Hz, 10 H), 1.43 (s, 3 H), 1.25 (d, *J* = 12.2 Hz, 6 H), 1.11 (d, *J* = 11.3 Hz, 6 H), 0.90 (s, 1 H), 0.35 (s, 3 H). ^13 ^C NMR (75 MHz, CDCl_3_), *δ*: 178.33, 177.21, 172.36, 170.23, 164.73, 146.01, 135.93, 133.95, 129.29, 128.49, 127.37, 127.10, 119.57, 118.00, 116.97, 52.78, 52.33, 45.03, 44.21, 43.01, 40.44, 39.26, 38.14, 37.78, 36.26, 34.72, 33.53, 33.45, 31.59, 30.79, 30.68, 29.97, 29.34, 28.64, 21.81, 18.08, 10.25. ESI-HRMS (*m/z*): calcd. for C_39_H_50_NO_5_^+^ [M + H]^+^: 612.3684; found: 612.3689. Purity: 100% by HPLC (A: H_2_O; B: acetonitrile, graded: 50–100%), *t*_R_ 17.247 min, λ: 400 nm.

#### 3-Hydroxy-9β,13α-dimethyl-2-oxo-24,25,26-trinoroleana-1(10),3,5,7-tetraen-29-oic amide, N-(S)-methyl 3-phenylpropanoate (8)

Red powder; yield, 72%; m.p. 132–134 °C; ^1 ^H NMR (300 MHz, CDCl_3_), *δ*: 7.25 (m, 2 H), 7.05(m, 4 H), 6.53 (s, 1 H), 6.34 (d, *J* = 6.9 Hz, 1 H), 6.06 (d, *J* = 6.3 Hz, 1 H), 4.64 (d, *J* = 6.4 Hz, 1 H), 3.71 (s, 3 H), 3.04 (dd, *J* = 14.3, 5.8 Hz, 2 H), 2.34 (s, 1 H), 2.24 (s, 3 H), 1.80 (m, 2 H), 1.61 (m, 12 H), 1.43 (s, 3 H), 1.27 (s, 3 H), 1.11 (d, *J* = 11.3 Hz, 6 H), 0.95 (s, 1 H), 0.35 (s, 3 H). ^13 ^C NMR (75 MHz, CDCl_3_), *δ*: 178.29, 176.04, 174.18, 170.23, 164.69, 146.01, 134.01, 130.15, 129.24, 128.55, 127.34, 127.18, 119.57, 118.06, 116.97, 53.78, 52.33, 45.03, 44.40, 42.98, 40.15, 39.30, 38.20, 37.89, 36.26, 34.72, 33.53, 33.30, 31.47, 30.80, 30.68, 29.98, 29.34, 28.63, 21.77, 18.24, 10.27. ESI-HRMS (*m/z*): calcd for C_39_H_50_NO_5_^+^ [M + H]^+^: 612.3684; found: 612.3686. Purity: 100% by HPLC (A: H_2_O; B: acetonitrile, graded: 50–100%), *t*_R_ 17.140 min, λ: 400 nm.

#### 3-Hydroxy-9β,13α-dimethyl-2-oxo-24,25,26-trinoroleana-1(10),3,5,7-tetraen-29-oic amide, N-(S)-dimethyl pentanedioate (9)

Red powder; yield, 60%; m.p. 124–126 °C; ^1 ^H NMR (300 MHz, CDCl_3_), *δ*: 7.00 (d, *J* = 6.8 Hz, 1 H), 6.65 (d, *J* = 6.9 Hz, 1 H), 6.52 (s, 1 H), 6.33 (d, *J* = 7.3 Hz, 1 H), 4.42 (d, *J* = 5.2 Hz, 1 H), 3.67 (d, *J* = 6.4 Hz, 6 H), 2.37 (dd, *J* = 19.5, 12.2 Hz, 3 H), 2.20 (s, 3 H), 2.12 (m, 3 H), 2.00 (m, 2 H), 1.83 (m, 3 H), 1.61 (s, 8 H), 1.43 (s, 3 H), 1.28–1.12 (m, 9 H), 0.82 (d, *J* = 18.1 Hz, 1 H), 0.54 (s, 3 H). ^13 ^C NMR (75 MHz, CDCl_3_), *δ*: 178.35, 178.04, 173.57, 172.63, 170.07, 164.71, 146.02, 133.98, 127.40, 119.51, 118.06, 116.99, 52.51, 51.91, 51.62, 45.05, 44.31, 42.99, 40.43, 39.32, 38.13, 36.30, 34.79, 33.73, 33.39, 31.62, 30.82, 30.76, 30.00, 29.94, 29.42, 28.67, 27.10, 21.82, 18.17, 10.25. ESI-HRMS (*m/z*): calcd. for C_36_H_50_NO_7_^+^ [M + H]^+^: 608.3582; found: 608.3571. Purity: 100% by HPLC (A: H_2_O; B: acetonitrile, graded: 50–100%), *t*_R_ 14.173 min, λ: 423 nm.

#### 3-Hydroxy-9β,13α-dimethyl-2-oxo-24,25,26-trinoroleana-1(10),3,5,7-tetraen-29-oic amide, N-(R)-methyl 2-amino-3–(1 H-imidazol-4-yl)propanoate (10)

Red powder; yield, 31%; m.p. 141–143 °C; ^1 ^H NMR (300 MHz, CDCl_3_), *δ*: 7.73 (d, *J* = 5.6 Hz, 1 H), 7.49 (s, 1 H), 7.07 (d, *J* = 7.1 Hz, 1 H), 6.85 (d, *J* = 20.6 Hz, 2 H), 6.49 (s, 1 H), 6.35 (m, 1 H), 5.02–4.87 (m, 1 H), 4.48–4.17 (m, 2 H), 3.84 (s, 2 H), 3.68 (s, 3 H), 2.67 (d, *J* = 9.3 Hz, 1 H), 2.23 (s, 3 H), 1.42(m, 3 H), 1.27 (m, 12 H), 1.24 (s, 3 H), 1.12 (d, *J* = 11.3 Hz, 6 H), 0.90 (s, 1 H), 0.50 (s, 3 H). ESI-HRMS (*m/z*): calcd for C_37_H_50_N_3_O_5_^+^ [M + H]^+^: 616.3745; found: 617.3752. Purity: 100% by HPLC (A: H_2_O; B: acetonitrile, graded: 50–100%), *t*_R_ 13.113 min, λ: 423 nm.

#### 3-Hydroxy-9β,13α-dimethyl-2-oxo-24,25,26-trinoroleana-1(10),3,5,7-tetraen-29-oic amide, N-(R)-methyl -3-(1 H-indol-2-yl)propanoate (11)

Red powder; yield, 46%; m.p. 136–138 °C; ^1 ^H NMR (300 MHz, CDCl_3_), *δ*: 8.37 (s, 1 H), 7.54 (d, *J* = 6.9 Hz, 1 H), 7.39 (d, *J* = 7.4 Hz, 1 H), 7.19 (d, *J* = 8.0 Hz, 1 H), 7.13 (d, *J* = 7.6 Hz, 1 H), 6.98 (s, 3 H), 6.53 (s, 1 H), 6.41–6.27 (m, 2 H), 4.64 (s, 1 H), 3.65 (s, 3 H), 3.25 (s, 2 H), 2.30 (d, *J* = 14.5 Hz, 1 H), 2.20 (s, 3 H), 2.00 (s, 2 H), 1.82 (d, *J* = 13.1 Hz, 2 H), 1.63 (s, 10 H), 1.40 (s, 3 H), 1.23 (d, *J* = 17.8 Hz, 9 H), 1.06 (s, 3 H), 0.87 (d, *J* = 6.1 Hz, 1 H), 0.44 (s, 3 H). ^13 ^C NMR (75 MHz, CDCl_3_), *δ*: 178.36, 177.55, 172.74, 170.64, 164.97, 146.03, 136.10, 134.34, 127.71, 127.29, 122.76, 122.25, 119.69, 119.37, 118.32, 118.02, 117.23, 121.40, 110.26, 53.69, 52.30, 45.01, 44.27, 43.02, 40.25, 39.24, 38.20, 36.27, 34.74, 33.53, 33.35, 31.59, 31.00, 30.68, 29.89, 29.25, 28.59, 27.45, 21.73, 18.04, 10.27. ESI-HRMS (*m/z*): calcd for C_41_H_51_N_2_O_5_^+^ [M + H]^+^: 651.3792; found: 651.3779. Purity: 100% by HPLC (A: H_2_O; B: acetonitrile, graded: 50–100%), *t*_R_ 15.893 min, λ: 400 nm.

#### General procedure for the synthesis of compound **12**–**24**

A mixture of celastrol (45 mg, 0.1 mmol), NaHCO_3_ (25 mg, 0.3 mmol), and halide derivatives (0.12 mmol) in DMF (4.0 ml) was stirred at 20 °C overnight. After confirming the progress of the reaction by thin-layer chromatography, the reaction mixture was transferred to 5 ml of water with 5 ml EA. The EA layer was washed with water (5 ml × 3) and saline (5 ml × 3) and dried using anhydrous MgSO_4_. The mixture was then purified by normal-phase column chromatography (PE:EA = 10:1) to obtain the target compounds **12**.

#### 3-Hydroxy-9β,13α-dimethyl-2-oxo-24,25,26-trinoroleana-1(10),3,5,7-tetraen-29-oicacid-(N-phenylacetamiden-1-yl)ethanone (12)

Red powder; yield, 38%; m.p. 90–92 °C; ^1 ^H NMR (300 MHz, CDCl_3_), *δ*: 7.70 (s, 1 H), 7.51 (d, *J* = 7.5 Hz, 2 H), 7.37 (t, *J* = 7.7 Hz, 2 H), 7.17 (s, 1 H), 6.98 (d, *J* = 13.8 Hz, 1 H), 6.44 (s, 1 H), 6.34 (d, *J* = 6.9 Hz, 1 H), 4.66 (d, *J* = 15.2 Hz, 1 H), 4.53 (d, *J* = 15.4 Hz, 1 H), 2.48 (d, *J* = 15.5 Hz, 1 H), 2.22 (s, 3 H), 2.06 (s, 2 H), 1.75 (s, 3 H), 1.53–1.75 (m, 8 H), 1.44 (s, 3 H), 1.35 (s, 3 H), 1.29 (s, 3 H), 1.16 (s, 3 H), 0.88 (d, *J* = 6.7 Hz, 1 H), 0.56 (s, 3 H). ^13 ^C NMR (75 MHz, CDCl_3_), *δ*: 178.35, 176.91, 169.02, 164.40, 164.01, 146.01, 136.69, 133.70, 129.23, 127.54, 125.02, 119.85, 119.80, 119.67, 118.24, 116.94, 63.26, 44.93, 44.35, 42.25, 42.77, 40.65, 39.42, 38.24, 36.31, 34.74, 33.39, 32.78, 31.58, 31.07, 30.54, 30.00, 29.90, 28.56, 21.62, 18.80, 10.25. ESI-HRMS (*m/z*): calcd for C_37_H_45_NNaO_5_^+^ [M + Na]^+^: 606.3190; found: 606.3180. Purity: 98.047% by HPLC (A: H_2_O; B: acetonitrile, graded: 50–100%), *t*_R_ 17.993 min, λ: 220 nm.

#### 3-Hydroxy-9β,13α-dimethyl-2-oxo-24,25,26-trinoroleana-1(10),3,5,7-tetraen-29-oic acid-(4-chlorophenylamino)ethanone (13)

Red powder; yield, 42%; m.p. 110–112 °C; ^1 ^H NMR (300 MHz, CDCl_3_), *δ*: 7.68 (s, 2 H), 7.46 (d, *J* = 8.9 Hz, 2 H), 7.00 (d, *J* = 10.2 Hz, 2 H), 6.47 (s, 1 H), 6.34 (d, *J* = 7.7 Hz, 1 H), 4.66 (d, *J* = 15.8 Hz, 1 H), 4.50 (d, *J* = 15.0 Hz, 1 H), 2.48 (d, *J* = 16.7 Hz, 1 H), 2.22 (s, 3 H), 1.82–1.57 (m, 12 H), 1.46 (s, 3 H), 1.37–1.27 (m, 9 H), 1.16 (s, 3 H), 0.98 (s, 1 H), 0.55 (s, 3 H). ^13 ^C NMR (75 MHz, CDCl_3_), *δ*: 178.32, 177.13, 169.27, 164.88, 164.52, 146.02, 135.38, 133.92, 129.91, 129.2, 127.49, 121.80, 119.58, 118.26, 117.73, 63.17, 44.95, 44.17, 42.81, 40.64, 39.38, 38.28, 36.26, 34.68, 33.40, 32.81, 31.56, 31.01, 30.54, 29.94, 29.72, 28.54, 21.61, 18.85, 10.30. ESI-HRMS (*m/z*): calcd for C_37_H_44_ClNNaO_4_^+^[M + Na]^+^: 640.3800; found: 640.3789. Purity: 100% by HPLC (A: H_2_O; B: acetonitrile, graded: 50–100%), *t*_R_ 13.460 min, λ: 423 nm.

#### 3-Hydroxy-9β,13α-dimethyl-2-oxo-24,25,26-trinoroleana-1(10),3,5,7-tetraen-29-oic acid-(2-fluorophenylamino)ethanone (14)

Red powder; yield, 28%; m.p. 118–122 °C; ^1 ^H NMR (300 MHz, CDCl_3_), *δ*: 8.38 (s, 1 H), 8.09 (s, 1 H), 7.17–7.11 (m, 2 H), 7.04–6.96 (m, 2 H), 6.45 (s, 1 H), 6.35 (d, *J* = 7.6 Hz, 1 H), 4.74–4.64 (m, 1 H), 4.55 (t, *J* = 8.4 Hz, 1 H), 2.49 (d, *J* = 14.9 Hz, 1 H), 2.22 (s, 3 H), 2.12–1.55 (m, 14 H), 1.45 (s, 3 H), 1.35 (s, 3 H), 1.29 (s, 3 H), 1.16 (s, 3 H), 0.87 (s, 1 H), 0.57 (s, 3 H). ^13 ^C NMR (75 MHz, CDCl_3_), *δ*: 178.36, 176.74, 169.13, 164.86, 164.44, 147.73, 146.00, 133.74, 127.53, 125.49, 125.36, 124.89, 121.44, 119.66, 118.24, 116.97, 114.91, 114.66, 63.21, 44.93, 44.24, 42.79, 42.79, 40.68, 39.43, 38.26, 36.30, 34.73, 33.40, 32.67, 31.58, 31.12, 30.53, 29.98, 29.84, 28.57, 21.64, 18.79, 10.24. ESI-HRMS (*m/z*): calcd for C_37_H_44_FNNaO_4_^+^[M + Na]^+^: 624.3096; found: 624.3089. Purity: 100% by HPLC (A: H_2_O; B: acetonitrile, graded: 50–100%), *t*_R_ 18.900 min, λ: 423 nm.

#### 3-Hydroxy-9β,13α-dimethyl-2-oxo-24,25,26-trinoroleana-1(10),3,5,7-tetraen-29-oicacid-N-(2-methoxyphenyl)acetamide (15)

Red powder; yield, 50%; m.p. 114–116 °C; ^1 ^H NMR (300 MHz, CDCl_3_), *δ*: 8.53 (s, 1 H), 8.40 (d, *J* = 7.8 Hz, 1 H), 7.15–7.08 (m, 1 H), 7.00 (d, *J* = 8.1 Hz, 2 H), 6.92 (d, *J* = 8.0 Hz, 1 H), 6.46 (s, 1 H), 6.35 (s, 1 H), 4.59 (dd, *J* = 36.9, 15.6 Hz, 2 H), 3.91 (s, 3 H), 2.52 (d, *J* = 15.7 Hz, 1 H), 2.22 (s, 3 H), 2.07–1.59 (m, 14 H), 1.45 (s, 3 H), 1.36 (s, 3 H), 1.29 (s, 3 H), 1.17 (s, 3 H), 0.89 (m, 1 H), 0.57 (s, 3 H). ^13 ^C NMR (75 MHz, CDCl_3_), *δ*: 178.36, 176.67, 169.96, 164.53, 164.41, 147.73, 146.01, 133.70, 126.67, 124.40, 124.31, 121.39, 119.76, 119.69, 118.22, 116.95, 109.91, 63.26, 55.61, 44.95, 44.31, 42.77, 40.63, 39.43, 38.25, 36.41, 34.69, 33.41, 32.68, 31.62, 33.11, 30.55, 29.91, 29.72, 28.56, 21.63, 18.74, 10.25. ESI-HRMS (*m/z*): calcd for C_38_H_47_NNaO_6_^+^ [M + Na]^+^: 636.3296; found: 636.3284. Purity: 100% by HPLC (A: H_2_O; B: acetonitrile, graded: 50–100%), *t*_R_ 19.320 min, λ: 423 nm.

#### 3-Hydroxy-9β,13α-dimethyl-2-oxo-24,25,26-trinoroleana-1(10),3,5,7-tetraen-29-oic acid-(4-ethylpiperazin-1-yl)ethanone (16)

Red powder; yield, 51%; m.p. 100–102 °C; ^1 ^H NMR (300 MHz, CDCl_3_), *δ*: 7.03 (s, 1 H), 6.56 (s, 1 H), 6.37 (d, *J* = 7.1 Hz, 1 H), 4.73 (m, H-1’b, 1 H), 4.52 (m, H-1’a, 1 H), 3.69 (s, 2 H), 3.44 (s, 2 H), 2.48 (m, 7 H), 2.23 (s, 3 H), 2.08–2.21 (m,5 H), 1.60 (m, 8 H), 1.47 (s, 3 H), 1.34 (s, 3 H), 1.28 (s, 3 H), 1.11 (m, 6 H), 1.00 (m, 1 H), 0.55 (s, 3 H). ^13 ^C NMR (75 MHz, CDCl_3_), *δ*: 178.31, 177.78, 170.40, 164.88, 164.74, 146.30, 134.19, 127.39, 119.57, 118.19, 117.27, 62.68, 52.43, 52.17, 51.98, 45.08, 44.24, 42.97, 40.49, 39.41, 38.34, 36.35, 34.68, 33.59, 32.76, 31.58, 30.82, 30.57, 29.81, 29.58, 28.67, 21.61, 18.76, 11.49, 10.26. ESI-HRMS (*m/z*): calcd. for C_37_H_52_N_2_N_a_O_5_^+^ [M + Na]^+^: 627.3768; found: 627.3765. Purity: 100% by HPLC (A: H_2_O; B: acetonitrile, graded: 50–100%), *t*_R_ 14.953 min, λ: 423 nm.

#### 3-Hydroxy-9β,13α-dimethyl-2-oxo-24,25,26-trinoroleana-1(10),3,5,7-tetraen-29-oicacid-(4-phenylpiperazin-1-yl)ethanone (17)

Red powder; yield, 52%; m.p. 118–120 °C; ^1 ^H NMR (300 MHz, CDCl_3_), *δ*: 7.32 (s, 2 H), 7.04 (d, *J* = 7.1 Hz, 1 H), 6.99 (s, 1 H), 6.92 (d, *J* = 8.1 Hz, 2 H), 6.57 (s, 1 H), 6.37 (d, *J* = 7.2 Hz, 1 H), 4.76 (s, 1 H), 4.58 (s, 1 H), 3.65 (d, *J* = 69.0 Hz, 4 H), 3.18 (d, *J* = 5.4 Hz, 4 H), 2.52 (d, *J* = 16.1 Hz, 1 H), 2.23 (s, 3 H), 1.61–2.11 (m, 14 H), 1.48 (s, 3 H), 1.36 (s, 3 H), 1.29 (s, 3 H), 1.13 (s, 3 H), 1.03–0.98 (m, 1 H), 0.57 (s, 3 H). ^13 ^C NMR (75 MHz, CDCl_3_), *δ*: 178.31, 177.77, 170.35, 164.86, 150.78, 146.02, 134.17, 129.27, 127.40, 120.76, 119.57, 118.19, 117.24, 116.78, 60.72, 50.88, 49.37, 45.08, 44.49, 44.25, 42.96, 40.50, 39.40, 38.35, 36.35, 34.67, 33.59, 32.76, 31.58, 30.83, 30.58, 29.84, 29.59, 28.67, 21.62, 18.77, 10.27. ESI-HRMS (*m/z*): calcd for C_41_H_52_N_2_NaO_5_^+^ [M + Na]^+^: 675.3768; found: 675.3758. Purity: 100% by HPLC (A: H_2_O; B: acetonitrile, graded: 50–100%), *t*_R_ 19.007 min, λ: 423 nm.

#### 3-Hydroxy-9β,13α-dimethyl-2-oxo-24,25,26-trinoroleana-1(10),3,5,7-tetraen-29-oic acid-(4-benzylpiperazin-1-yl)ethanone (18)

Red powder; yield, 45%; m.p. 110–112 °C; ^1 ^H NMR (300 MHz, CDCl_3_), *δ*: 7.32 (m, 4 H), 7.04 (d, *J* = 6.8 Hz, 1 H), 6.97 (s, 1 H), 6.55 (s, 1 H), 6.36 (d, *J* = 6.9 Hz, 1 H), 4.73 (d, *J* = 14.3 Hz, 1 H), 4.48 (d, *J* = 14.4 Hz, 1 H), 3.57 (m, *J* = 33.6 Hz, 4 H), 3.36 (s, 2 H), 2.44 (s, 7 H), 2.23 (s, 3 H), 1.60–1.86 (m, 14 H), 1.47 (s, 3 H), 1.34 (s, 3 H), 1.28 (s, 3 H), 1.12 (s, 3 H), 0.87 (m, 1 H), 0.56 (s, 3 H). ^13 ^C NMR (75 MHz, CDCl_3_), *δ*: 178.30, 177.75, 170.32, 164.85, 164.70, 146.00, 137.46, 134.13, 129.09, 128.35, 127.39, 127.32, 119.56, 118.16, 117.17, 62.81, 60.76, 52.84, 52.47, 45.07, 44.46, 44.25, 42.96, 41.89, 40.49, 39.40, 38.33, 36.35, 34.69, 33.58, 32.77, 31.58, 30.83, 30.57, 29.81, 29.59, 28.67, 21.63, 18.75, 10.26. ESI-HRMS (*m/z*): calcd for C_42_H_54_N_2_N_a_O_5_^+^ [M + Na]^+^: 689.3925; found: 689.3920. Purity: 100% by HPLC (A: H_2_O; B: acetonitrile, graded: 50–100%), *t*_R_ 19.833 min, λ: 423 nm.

#### 3-Hydroxy-9β,13α-dimethyl-2-oxo-24,25,26-trinoroleana-1(10),3,5,7-tetraen-29-oic acid-1-morpholinoethanone (19)

Red powder; yield, 46%; m.p. 112–114 °C;^1 ^H NMR (300 MHz, CDCl_3_), *δ*: 7.04 (d, *J* = 6.7 Hz, 1 H), 6.55 (s, 1 H), 6.37 (d, *J* = 7.3 Hz, 1 H), 4.74 (d, *J* = 14.2 Hz, 1 H), 4.48 (d, *J* = 14.4 Hz, 1 H), 3.84–3.71 (m, 8 H), 3.38 (s, 2 H), 2.50 (d, *J* = 16.6 Hz, 1 H), 2.23 (s, 3 H), 1.60–1.55 (m, 8 H), 1.47 (s, 3 H), 1.35 (s, 4 H), 1.26 (s, 3 H), 1.24 (s, 3 H), 1.13 (s, 3 H), 0.88 (d, *J* = 7.5 Hz, 1 H), 0.55 (s, 3 H). ^13 ^C NMR (75 MHz, CDCl_3_), *δ*: 178.30, 177.76, 170.30, 165.04, 164.85, 146.01, 134.15, 127.40, 119.56, 118.19, 117.24, 66.74, 60.61, 58.41, 45.06, 44.23, 42.95, 42.06, 40.49, 39.38, 38.35, 36.34, 34.65, 33.57, 32.75, 30.82, 30.57, 29.81, 29.70, 29.59, 28.66, 21.62, 18.77, 18.45, 10.27. ESI-HRMS (*m/z*): calcd for C_35_H_47_NNaO_6_^+^ [M + Na]^+^: 600.3296; found: 600.3285. Purity: 100% by HPLC (A: H_2_O; B: acetonitrile, graded: 50–100%), *t*_R_ 15.100 min, λ: 423 nm.

#### 3-Hydroxy-9β,13α-dimethyl-2-oxo-24,25,26-trinoroleana-1(10),3,5,7-tetraen-29-oic acid-(1-piperidin-1-yl)ethanone (20)

Red powder; yield, 56%; m.p. 94–96 °C; ^1 ^H NMR (300 MHz, CDCl_3_), *δ*: 7.02 (d, *J* = 7.0 Hz, 1 H), 6.96 (s, 1 H), 6.54 (s, 1 H), 6.34 (d, *J* = 7.1 Hz, 1 H), 4.72 (d, *J* = 14.2 Hz, 1 H), 4.46 (d, *J* = 14.3 Hz, 1 H), 3.75–3.69 (m,4 H), 3.26 (m, 2 H), 2.49 (d, *J* = 16.2 Hz, 1 H), 2.21 (s, 3 H), 1.54–1.84 (m, 14 H), 1.45 (s, 3 H), 1.26 (m, 6 H), 1.22 (s, 3 H), 1.10 (s, 3 H), 0.86 (d, *J* = 6.4 Hz, 1 H), 0.54 (s, 3 H). ^13 ^C NMR (75 MHz, CDCl_3_), *δ*: 178.31, 177.78, 170.42, 164.89, 164.48, 146.01, 134.17, 127.38, 119.55, 118.16, 117.19, 61.60, 58.41, 45.08, 44.26, 42.97, 40.47, 39.40, 38.33, 36.37, 34.70, 33.58, 32.77, 31.58, 30.84, 30.57, 29.83, 29.58, 28.68, 26.27, 25.33, 24.37, 21.63, 18.73, 18.45, 10.26. ESI-HRMS (*m/z*): calcd for C_36_H_50_NNaO_5_^+^ [M + Na]^+^: 598.3503; found: 598.3493. Purity: 100% by HPLC (A: H_2_O; B: acetonitrile, graded: 50–100%), *t*_R_ 17.973 min, λ: 400 nm.

#### 3-Hydroxy-9β,13α-dimethyl-2-oxo-24,25,26-trinoroleana-1(10),3,5,7-tetraen-29-oic acid-(pyrrolidin-1-yl)ethanone (21)

Red powder; yield, 54%; m.p. 109–111 °C; ^1 ^H NMR (300 MHz, CDCl_3_), *δ*: 7.04 (d, *J* = 7.1 Hz, 1 H), 6.56 (s, 1 H), 6.37 (d, *J* = 4.4 Hz, 1 H), 4.62 (d, *J* = 14.5 Hz, 1 H), 4.39 (d, *J* = 14.6 Hz, 1 H), 3.35 (m, 4 H), 2.52 (d, *J* = 15.9 Hz, 1 H), 2.23 (d, *J* = 2.8 Hz, 3 H), 1.47–1.96 (m, 18 H), 1.47 (s, 3 H), 1.34 (s, 3 H), 1.28 (s, 3 H), 1.12 (s, 3 H), 0.90 (s, 1 H), 0.56 (s, 3 H). ^13 ^C NMR (75 MHz, CDCl_3_), *δ*: 178.30, 177.89, 170.55, 167.22, 164.90, 146.01, 134.27, 127.36, 119.54, 118.17, 117.26, 62.90, 61.31, 45.89, 45.10, 44.25, 43.01, 40.48, 39.42, 38.33, 36.37, 34.71, 33.58, 32.79, 31.59, 30.80, 30.57, 29.80, 29.57, 28.68, 26.07, 23.88, 21.62, 18.70, 10.26. ESI-HRMS (*m/z*): calcd for C_35_H_47_NNaO_5_^+^[M + Na]^+^: 584.3346; found: 584.3338. Purity: 100% by HPLC (A: H_2_O; B: acetonitrile, graded: 50–100%), *t*_R_ 15.853 min, λ: 400 nm.

#### 3-Hydroxy-9β,13α-dimethyl-2-oxo-24,25,26-trinoroleana-1(10),3,5,7-tetraen-29-oic acid-(diethylamino-1-yl))ethanone (22)

Red powder; yield, 33%; m.p. 98–100 °C; ^1 ^H NMR (300 MHz, CDCl_3_), *δ*: 7.14–6.96 (m, 2 H), 6.56 (s, 1 H), 6.37 (d, *J* = 7.1 Hz, 1 H), 4.71 (d, *J* = 14.2 Hz, 1 H), 4.48 (d, *J* = 14.1 Hz, 1 H), 3.38 (dd, *J* = 14.3, 7.2 Hz, 2 H), 3.22 (dd, *J* = 14.4, 7.4 Hz, 2 H), 2.53 (d, *J* = 15.7 Hz, 1 H), 2.23 (s, 3 H), 1.69–2.16 (m, 8 H), 1.60 (s, 3 H), 1.47 (s, 3 H), 1.36 (s, 3 H), 1.29 (s, 3 H), 1.19 (s, 1 H), 1.15 (m, 7 H), 1.00 (m, 2 H), 0.89 (s, 1 H), 0.57 (s, 3 H). ^13 ^C NMR (75 MHz, CDCl_3_), *δ*: 178.31, 177.91, 170.54, 165.34, 164.93, 146.00, 134.25, 127.35, 119.54, 118.16, 117.22, 60.68, 45.10, 44.24, 43.00, 40.78, 40.45, 40.27, 39.41, 38.35, 36.37, 34.71, 33.57, 32.80, 31.59, 30.79, 30.57, 29.83, 29.57, 28.67, 21.63, 18.71, 14.18, 12.94, 10.28. ESI-HRMS (*m/z*): calcd for C_35_H_49_NNaO_5_^+^ [M + Na]^+^: 586.3503; found: 586.3507. Purity: 100% by HPLC (A: H_2_O; B: acetonitrile, graded: 50–100%), *t*_R_ 17.320 min, λ: 423 nm.

#### 3-Hydroxy-9β,13α-dimethyl-2-oxo-24,25,26-trinoroleana-1(10),3,5,7-tetraen-29-oic acid-(3-(4-chlorobenzyl)-3 H-1,2,3-triazol-4-yl)methyl (23)

Red powder; yield, 53%; m.p. 98–100 °C; ^1 ^H NMR (300 MHz, CDCl_3_), *δ*: 7.48–7.29 (m, 5 H), 7.13 (d, *J* = 8.1 Hz, 1 H), 7.04 (d, *J* = 6.9 Hz, 1 H), 6.54 (s, 1 H), 6.35 (d, *J* = 7.1 Hz, 1 H), 5.46 (d, *J* = 27.5 Hz, 2 H), 5.08 (d, *J* = 6.4 Hz, 2 H), 2.40 (s, 1 H), 2.22 (s, 3 H), 1.76 (m, 12 H), 1.45 (s, 3 H), 1.26 (d, *J* = 4.1 Hz, 3 H), 1.12 (d, *J* = 15.3 Hz, 6 H), 1.00 (s, 1 H), 0.44 (s, 3 H). ^13 ^C NMR (75 MHz, CDCl_3_), *δ*: 178.27, 178.11, 170.18, 164.81, 146.03, 143.01, 134.77, 134.14, 132.96, 129.31, 129.25, 127.37, 123.47, 119.53, 118.15, 117.22, 57.22, 53.32, 44.99, 44.13, 42.90, 40.39, 39.34, 38.28, 36.30, 34.60, 33.44, 32.77, 31.51, 30.62, 30.53, 29.71, 29.49, 28.60, 21.59, 18.54, 10.27. ESI-HRMS (*m/z*): calcd for C_39_H_46_ClN_3_O_4_^+^ [M + H]^+^: 656.3250; found: 656.3233. Purity: 92.00% by HPLC (A: H_2_O; B: acetonitrile, graded: 50–100%), *t*_R_ 19.337 min, λ: 423 nm.

#### 3-Hydroxy-9β,13α-dimethyl-2-oxo-24,25,26-trinoroleana-1(10),3,5,7-tetraen-29-oic acid-(3-(4-fluorobenzyl)-3 H-1,2,3-triazol-4-yl)methyl (24)

Red powder; yield, 55%; m.p. 102–104 °C; ^1 ^H NMR (300 MHz, CDCl_3_) *δ*: 7.41 (s, 1 H), 7.19 (s, 2 H), 7.05 (t, *J* = 6.6 Hz, 5 H), 6.55 (s, 1 H), 6.35 (d, *J* = 6.7 Hz, 1 H), 5.42 (s, 1 H), 5.32 (d, *J* = 2.2 Hz, 1 H), 5.15–5.03 (m, 2 H), 2.43 (d, *J* = 15.0 Hz, 1 H), 2.23 (s, 3 H), 1.77–1.52 (m, 12 H), 1.45 (s, 3 H), 1.26 (s, 3 H), 1.15 (s, 3 H), 1.10 (s, 3 H), 1.01 (s, 1 H), 0.44 (s, 3 H). ^13 ^C NMR (75 MHz, CDCl_3_), *δ*: 178.28, 178.14, 170.14, 164.81, 146.02, 142.97, 134.09, 129.90, 129.78, 127.39, 123.39, 119.54, 118.13, 117.13, 116.27, 115.98, 57.24, 53.34, 45.00, 44.14, 42.91, 40.40, 39.34, 38.29, 36.30, 34.62, 33.45, 32.77, 31.53, 30.53, 29.71, 29.50, 28.60, 21.60, 18.54, 13.75, 10.27. ESI-HRMS (*m/z*): calcd for C_39_H_47_FN_3_O_4_^+^ [M + H]^+^: 640.3545; found: 640.3543. Purity: 100% by HPLC (A: H_2_O; B: acetonitrile, graded: 50–100%), *t*_R_ 18.140 min, λ: 400 nm.

### Anticancer assay

The anti-proliferative activity of the title compounds against the a panel of eight different human cancer cell lines viz. gastric (AGS), differentiation of advanced gastric (MGC-803), colorectal (HCT-116), differentiation of Early gastric (SGC-7901), liver (BEL-7402), Lung (A549), liver (HepG2) and cervix (HeLa) cell lines were evaluated using a standard MTT-based colorimetric assay.

All cell lines were obtained from the Key Laboratory of Natural Resources and Functional Molecules of the Changbai Mountain (Yanbian University) and maintained in Dulbecco’s modified Eagle’s medium (DMEM) and RPMI Media 1640 (RPMI1640), supplemented with 10% foetal bovine serum (FBS) at 37 °C in a humidified atmosphere containing 5% CO_2._

### MTT assay

Cells were plated in 96-well plates at appropriate densities to ensure exponential growth throughout the experimental period (9 × 10^3^ cells per well), and then allowed to adhere for 24 h. Cells were then treated for 48 h with four serial concentrations (1, 10, 50, and 100 μM) of each compound. Taxol was used as a positive control. After 48 h of incubation, 10 μL of MTT solution were added to each well to a final concentration of 2 mg mL^−1^. Plates were then incubated for a further 4 h. After incubation, the MTT solution was removed and 150 μL of DMSO were added to each well for coloration. The plates were shaken vigorously for 10 min at room temperature to ensure complete solubilisation. The optometric density (OD) was read on a microplate reader (ELx800, BioTek, Highland Park, Winooski, VT, USA) at 492 nm, and the data were subsequently analysed. The percentage of cell growth inhibition was calculated from the following equation:

$$\text{Inhibitory rate}\left(\%\right)=[\text{1}-({\text{OD}}_{\text{treated}}-{\text{OD}}_{\text{blank}})/({\text{OD}}_{\text{control}}-{\text{OD}}_{\text{blank}})]\times \text{1}00.$$

### Analysis for cell cycle by flow cytometry

AGS cells were plated in 96-well plates (5.0 × 10^5^ cells per well) and incubated at 37 °C for 12 h. Exponentially growing cells were then incubated with compound **11** at different concentrations (0.5, 1.0, and 5.0 μM). After 48 h, untreated cells (control) or cells treated with compound **11** were centrifuged at 1000 rpm for 10 min, and then fixed in 70% ethanol at −20 °C for at least 24 h. The cells were subsequently resuspended in phosphate-buffered saline (PBS) containing 0.1 mg mL^−1^ RNase A and 5 μg mL^−1^ propidium iodide (PI). The cellular DNA content for the cell cycle distribution analysis was measured by flow cytometry using a FACS Calibur flow cytometer with Cell Quest software (Becton-Dickinson, Franklin Lakes, NJ), plotting at least 30 000 events per sample. The percentage of cells in the G1, S and G2 phases of the cell cycle were determined using the ModFit LT V4.0 software package (Verity Software, Topsham, ME).

#### Analysis for apoptosis by flow cytometry

Apoptosis was detected using an Apoptosis Detection Kit (Invitrogen, Eugene, OR). In brief, cells were cultured in 96-well plates (5.0 × 10^5^ cells per well) and incubated at 37 °C for 12 h. Cells with exponential growth were then incubated with compound **11** at different concentrations (0.1, 1.0, and 5.0 μM). Following 48 h of incubation, the cells were collected, washed twice with PBS and once with 1 × binding buffer, and then stained with 5 μM of annexin V-FITC and 2.5 μM of PI (5 mg mL^−1^) in 1 × binding buffer for 30 min at 20 °C in the dark. Apoptotic cells were enumerated using a FACSCalibur flow cytometer with Cell Quest software (Becton–Dickinson)

#### Docking study

The molecular docking study was performed using Discovery Studio (DS) 2017. The ligand and protein were prepared, hydrogen was added and water molecules were deleted by DS Server. The result of docking was treated with DS Client. In this study, the crystal structure of Casepase-3 complex (1GFW) was chosen for docking. The xyz coordinates (37.6468, 33.5653, 27.9733, radius 8.12325 Å) of protein residues were defined as the binding site sphere. The protocol, Dock Ligant (LibDock) was used to perform the docking. The output poses of the ligands generated were analysed based on the LibDockScore function.

## Results and discussion

### Chemistry

**Scheme 1.**
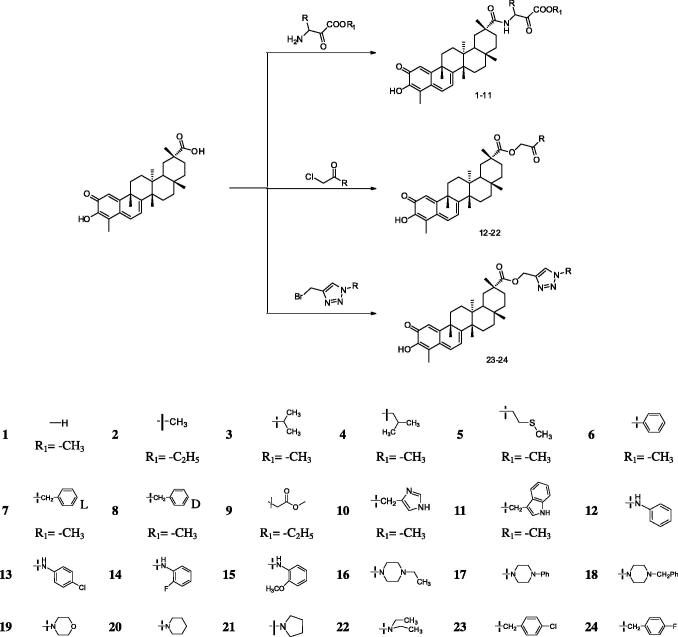
Synthesis of celastrol derivatives (1–24).

The reaction occurred mainly at the 20th position of celastrol (Scheme 1). Compounds **1**–**11** were obtained by treating celastrol with different amino acids under the catalysis of NaHCO_3_ in anhydrous DMF, with 26–61% yield. Compounds **12**–**22** were obtained by an amide condensation reaction catalysed by EDC·HCl, HOBt, and TEA in anhydrous CH_2_Cl_2_, with 33–56% yield. Compounds **23** and **24** were prepared by treating celastrol with phenylbromostannyl. The intermediate 4-(bromomethyl)-1-(4-substituted-benzyl)-1 *H*-1,2,3-triazole was formed by the interactions between 1-(azidomethyl)-4-substituted-benzene and propargyl bromide and CuSO_4_·5H_2_O and VC-Na, respectively.

### *In vitro* anticancer activity

All synthesised compounds were evaluated for their anticancer activities *in vitro* against AGS, MGC-803, SGC-7901, HCT-116, A549, HeLa, BEL-7402, and HepG-2 cell lines. The activity of celastrol was used as reference. Cells were allowed to proliferate in presence of the test compounds for 48 h, and the results are presented as IC_50_ values (Table 1). Most of the synthesised compounds showed highly significant antiproliferative effects. Among them, compounds **1**, **2**, **3**, **4**, **6**, **8**, **9**, **10**, **11**, **15**, **17**, **19**, **20**, **21**, **22**, and **24** showed potent inhibitory activities (IC_50_ ≤ 1 μM; for compounds **8**, **9**, **10**, **11**, **17**, **19**, **20**, **21**, **22**, and **24**, IC_50_ = 0.74, 0.72, 0.75, 0.44, 0.75, 0.68, 0.85, 0.88, 0.49, and 0.97 μM, respectively, against AGS cells; for compounds **15** and **22**, IC_50_ = 0.71, and 0.78 μM, respectively, against MGC-803 cells; for compounds **8**, **11**, **17**, **20**, and **22**, IC_50_ = 0.90, 0.78, 0.88, 0.84, and 0.85 μM, respectively, against HCT-116 cells; for compounds **1**, **3**, **6**, **8**, **11**, and **22**, IC_50_ = 0.89, 0.81, 0.60, 0.68, 0.71, 0.63, and 0.98 μM, respectively, against BEL-7402 cells; for compounds **8** and **17**, IC_50_ = 0.96 and 0.91 μM, respectively, against A549 cells; for compounds **3**, **17**, **20**, and **22**, IC_50_ = 0.73, 0.82, 0.75, and 0.62 μM, respectively, against HeLa cells; and for compounds **1**, **3**, **4**, **6**, **8**, **17**, **19**, **20**, and **22**, IC_50_ = 0.85, 0.66, 0.90, 0.88, 0.97, 0.76, 1.00, 0.91, and 0.84 μM, respectively, against HepG-2 cells,), which were greater than that of the reference celastrol.

**Table 1.** Anticancer effects of compounds **1**–**24** as analysed by the MTT assay.

	(μM) IC_50_							
Compd.	AGS	MGC-803	HCT-116	SGC-7901	BEL-7402	A549	HELA	HEPG-2
**1**	1.05	3.13	1.67	1.78	0.89	1.64	1.89	0.85
**2**	1.02	2.47	1.48	1.63	0.81	1.62	1.65	1.03
**3**	1.38	33.16	3.31	2.01	1.36	2.00	2.49	0.9
**4**	1.01	2.4	2.83	1.53	0.6	1.99	0.73	0.66
**5**	8.06	9.52	9.67	21.25	2.62	10.33	1.02	4.63
**6**	1.29	2.19	2.23	1.75	0.68	2.39	1.05	0.88
**7**	28.04	14.92	3.52	25.82	1.82	6.81	21.47	7.37
**8**	0.74	1.33	0.90	1.47	0.71	0.96	1.27	0.97
**9**	0.72	2.46	1.30	1.91	1.19	2.02	1.26	1.39
**10**	0.75	6.28	5.26	5.89	2.44	3.59	3.65	2.47
**11**	0.44	1.03	0.78	1.66	0.63	1.22	2.63	0.76
**12**	0.49	0.78	0.85	1.52	0.98	2.06	0.62	1.02
**13**	1.93	1.30	2.7	1.57	3.5	2.15	2.83	1.82
**14**	1.42	1.07	0.93	1.95	1.61	3.81	1.67	4.65
**15**	2.28	1.57	1.68	2.15	1.91	5.23	3.61	5.83
**16**	1.09	1.70	1.33	4.14	1.98	1.72	1.08	1.19
**17**	0.75	1.19	0.88	1.81	1.14	0.91	0.815	0.91
**18**	1.35	1.69	2.00	4.19	2.35	1.77	1.31	1.18
**19**	0.68	1.81	1.38	2.54	1.18	2.23	2.25	1.00
**20**	0.85	1.18	0.84	2.14	1.19	1.71	0.75	0.84
**21**	0.88	2.34	1.76	1.94	2.27	2.26	2.22	1.43
**22**	1.10	2.16	1.98	5.39	4.16	3.53	1.84	1.56
**23**	0.97	2.95	5.23	4.81	4.89	3.52	2.66	2.19
**24**	5.66	5.77	12.25	61.71	–	54.94	23.47	–
Celastrol	1.46	4.55	3.43	5.71	4.05	3.02	1.51	1.31

Using structure–activity relationship (SAR) studies, we attempted to demonstrate how the substituent at the 20th carboxylic acid position of celastrol affected its anticancer activity. Celastrol showed good antiproliferative activity. In particular, it showed remarkable antiproliferative effects against AGS, HeLa, and HepG-2 cell lines with IC_50_ values of 1.46, 1.51, and 1.31 μM, respectively. Compounds **1**–**4**, products of reaction with aliphatic amino acid esters, inhibited the proliferation of most of the tumour cell lines to a greater extent than did celastrol. In particular, the inhibitory activity increased significantly in BEL-7402 cell lines. This indicated that the introduction of hydrophobic groups strengthened the antitumor activity, although significant patterns were not established. By introducing a sulphur (S) atom, compound **5** showed lower inhibitory activity than did compound **4** in most of the tumour cell lines. Remarkably, it showed similar antiproliferative activity to that of compound **4** (IC_50_ 0.73 μM) against HeLa cell lines, with an IC_50_ value of 1.02 μM. Thus, we suspected that the S atoms played an important role in antiproliferation in HeLa cells. The screening results of compound **6**–**8** showed that compound **6** had lower antiproliferative activity than did compound **8**; however, compound 6 had better antiproliferative activity against the 8 cell lines tested than did compound **7**. Unexpectedly, the anticancer activity of compound **7** was much lower than that of compound **8**. This raised an uncertainty as to whether space configuration played a more fundamental role in inhibitory potential than did the length of the carbon chain of amino acids during substitution. Compounds **9**–**11** showed considerably higher antiproliferative activity against AGS and BEL-7402 cell lines. Moreover, compound **11** showed the highest anticancer activity against AGS cell lines. It also showed better anticancer activity against HCT-116, BEL-7402, and HepG-2 cell lines than the other compounds. Compound **12**–**15** showed anticancer activities of varying degrees. In general, introduction of aniline groups had a positive influence on the anticancer activity as compared to that of celastrol; however, their inhibitory activities decreased following the introduction of –Cl, –F, –OCH_3_ groups, respectively. Among compounds **16**–**18**, where the R-position was substituted with different piperazidine derivatives, compound **17**, which had a nitrogen (N) atom linked to the benzene ring directly, showed the highest anticancer activity. Compounds **16** and **18** showed lower activity, as the N atom was linked to the branched alkyl or the benzene ring indirectly. These results indicate that the inhibitory activity considerably improved when the N atom of piperazidine was linked to the benzene ring directly. Compounds **19**–**21**, heterocyclic compounds with an N atom, expressed higher inhibitory activities against AGS, MGC-803, SGC-7901, HCT-116, A549, and BEL-7402 cell lines than did celastrol. In particular, the IC_50_ values of compound **20** and **21** were 3-fold higher than that of celastrol against SGC-7901, HCT-116, and BEL-7402 cell lines. This result indicates that the activity of substituted groups was increasing in the order piperidine > morpholine, pyrrolidine. Compound **22** showed higher inhibitory activity than did celastrol (4.55, 3.43 μM) against MGC-7901 and HCT-116 cell lines with IC_50_ values of 2.16 and 1.98 μM, respectively. These data of compound **23** and **24** suggested that the introduction of 1,2,3-triazole linked to benzyl chloride had a minor influence on the anticancer activity of celastrol.

### Compound **11** induces AGS cell cycle arrest and apoptosis

**Figure 1.**
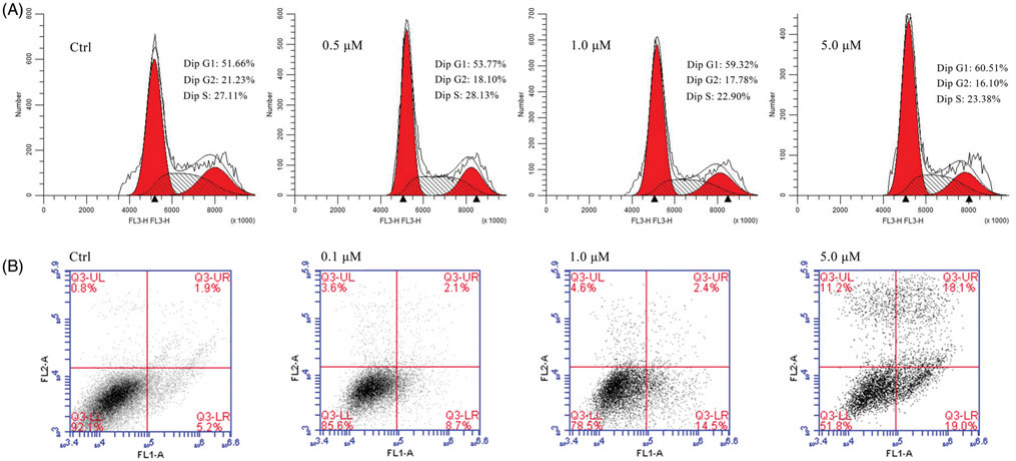
Cell cycle and apoptosis analysis of compound **11** in AGS cells.

In this study, flow cytometry was used to determine whether compound **11**-mediated inhibition of growth and proliferation was associated with apoptosis. AGS cell lines were treated with compound **11** at concentrations of 0.5, 1.0, and 5.0 μM for 48 h, and the results are shown in Figure 1(A). The proportion of cells in the G1 phase increased from 51.66% (control) to 53.77% (0.5 μM), 59.32(1.0 μM) and 60.51% (5.0 μM), and the proportion of cells in the G2 phase decreased from 21.23% (control) to 18.10% (0.5 μM), 17.70% (1.0 μM) and 16.10% (5.0 μM). Although the rate of G1 and G2 phase presented a trend of change with the increase of the concentration of compound **11**, it did not show significant difference. These results indicate that compound **11** may be weaker influence on cell cycle arrest. Next, cell apoptosis analysis was performed to determine whether compound **11** can induce apoptosis of cells. As shown in Figure 1(B), the early stage apoptosis rate of the control group was 5.2%. The early apoptosis rates gradually increased from 8.7%, 14.5% to 19.0% and the late stage apoptosis rates increased from 2.1%, 2.4%, to 18.1% after treatment with 0.1 μM, 1.0 μM, and 5.0 μM of compound **11**, respectively, for 48 h. This suggests that compound **11** induced apoptosis and caused a marked increase in apoptosis in a concentration-dependent manner.

**Figure 2.**
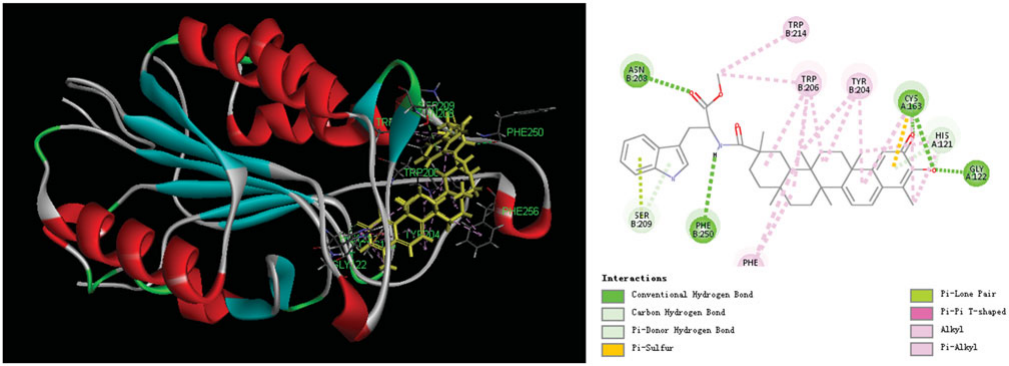
Computer modelling of compound **11** binding to Caspase-3 (1GFW). Compound **11** was colored in yellow.

### Docking analysis

Apoptosis signalling can be initiated either at the cell surface via a death receptor-induced signalling pathway or within the cell via the release of pro-apoptotic factors^22^. Fan et al. have reported that activation of caspases underlies the apoptotic process induced by celastrol^23^. In all probability, compound **11** as a celastrol derivative could induce apoptosis by inducing the activation of caspases. The caspase-3 plays a key role in the apoptotic pathway^24^. To confirm this speculation, in this text, docking simulation was performed to position compound **11** into the caspase-3 (1GFW) active site to determine the probable binding model (Figure 2). The result revealed that five conventional hydrogen bonds and three carbon hydrogen bonds are observed with residue His 121, Gly 122, Cys 163, Asn 208, and Phe 250. Also, the indole ring interacted with the residue Ser 209. This result gives some clue for the mechanism of compound **11** inducing apoptosis by inducing the activation of caspase-3. Therefore, these results indicate that the potent antiproliferative activity of compound **11** possibly involved in inducing apoptosis by inducing the activation of caspase-3.

## Conclusions

In summary, we designed and synthesised three series of celastrol derivatives (**1–24**), and evaluated their anticancer effects against eight cancer cell lines (AGS, MGC-803, SGC-7901, HCT-116, A549, HeLa, BEL-7402, and HepG-2). Most of the target compounds exhibited potent inhibitory activity *in vitro*, and the antiproliferative activity of these compounds was screened via the MTT assay. In particular, compound **11** exhibited excellent inhibitory activity against AGS cells, with an IC_50_ value of 0.44 μM. Moreover, it showed higher inhibitory activities against HCT-116, BEL-7402, and HepG-2 cell lines, with IC_50_ values of 0.78, 0.63, and 0.76 μM, respectively. The results of experiments on cell cycle arrest and apoptosis induced by compound **11** suggested that it induced apoptosis in AGS cells. Docking study also revealed the amino acids His-121, Gly122, Asn 208, and Phe 250 were found to be playing crucial role in the binding of compound **11** within the active site of caspase-3. Therefore, the modifications to the 20th position of celastrol in this study were helpful to improve its anticancer activity.
